# Migration of ingested sewing needle from within sigmoid colon to outside of the lumen

**DOI:** 10.12669/pjms.306.5423

**Published:** 2014

**Authors:** Mehmet Nuri Cevizci, Muhammet Demir, Berrin Demir, Ilknur Demir, Omer Kilic

**Affiliations:** 1 Mehmet Nuri Cevizci, MD, Department of Pediatric Surgery, Erzurum District Training and Research Hospital, Erzurum, Turkey.; 2 Muhammet Demir, MD, Department of Pediatric Surgery, Erzurum District Training and Research Hospital, Erzurum, Turkey.; 3 Berrin Demir, MD, Department of Radiology, Erzurum District Training and Research Hospital, Erzurum, Turkey.; 4 Ilknur Demir, MD, Department of Pediatrics, Erzurum District Training and Research Hospital, Erzurum, Turkey.; 5 Omer Kilic, MD, Department of Pediatric Infectious Diseases, Erzurum District Training and Research Hospital, Erzurum, Turkey.

**Keywords:** Sewing needle ingestion, Foreign body migration, Child

## Abstract

Foreign body ingestion is a frequently observed condition in children. However, migration of an ingested foreign body from the gastrointestinal tract toward any abdominal organ is extremely rare. We report herein a case of a 2-year-old female patient in whom an ingested sewing needle was palpable by rectal examination and was determined to have migrated from within the sigmoid colon to outside of the lumen. The needle was surgically removed. In cases of foreign body ingestion, both physical examination and radiological follow-up should be performed.

## INTRODUCTION

Foreign body ingestion is relatively frequently seen in children, particularly those between the ages of 6 months and 3 years.^1^ Although children most commonly ingest coins, a broad array of other foreign bodies can also be observed. Approximately 80% to 90% of ingested foreign bodies smoothly migrate from the gastrointestinal tract under close observation; 10% to 20% of these are removed by an endoscopic procedure, and ≤1% of cases require a surgical procedure due to complications such as obstruction, perforation, or fistula formation.^2^ Migration of an ingested foreign body from the gastrointestinal tract toward any abdominal organ is extremely rare. We report herein a case of a 2-year-old female patient in whom an ingested sewing needle was followed in another center for 3 months but was not spontaneously expelled. The sewing needle had migrated from within the sigmoid colon to outside of the lumen and was removed by a surgical procedure. This case is presented to emphasize the fact that, an ingested sharp foreign can migrate outside the lumen if not removed early. In addition physical examination and radiological follow-up are important in such cases to identify the complications.

## CASE REPORT

**Fig.1:**
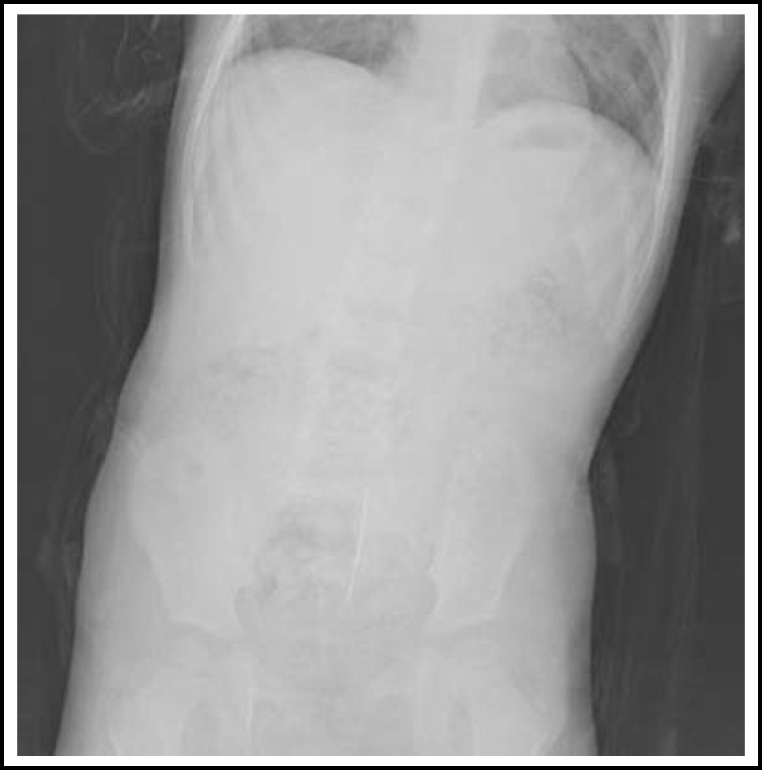
Abdominal radiograph shows a radiopaque foreign body in the lower abdomen

**Fig.2:**
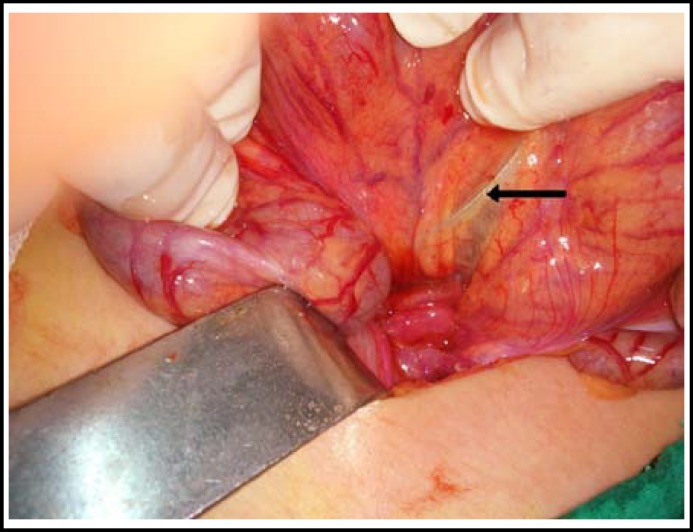
Intraoperative photograph of the sewing needle (arrow) in the subserosa of sigmoid colon

A 2-year-old female patient with history of sewing needle ingestion about three months back followed by mild intermittent abdominal pain. The child was followed in another hospital for but the foreign body was not expelled spontaneously. A plain abdominal X-ray on arrival showed a radiopaque foreign body (sewing needle) in the lower abdominal quadrant on the midline (Fig.1). The foreign body was palpable by rectal examination; however, it could not be observed by proctoscopy performed under general anesthesia. Thus, considering that the foreign body might have migrated outside of the lumen, a mini-laparotomy procedure was performed. The sewing needle was observed in the sigmoid mesocolon and removed by opening a small window into the mesocolon (Fig.2). Child had a recovery and no problems were observed during the 3-month follow-up of the patient.

## DISCUSSION

Foreign body (FB) ingestion is most frequently observed in children, especially those between the ages of 6 months and 3 years. About 40% of foreign body ingestions in children are not witnessed, and approximately half of those are asymptomatic.^3^ Most ingested foreign bodies pass through the gastrointestinal tract smoothly. Gastrointestinal tract perforation related to foreign bodies is observed in <1% of patients, frequently in the ileocecal and rectosigmoid regions. An urgent examination of cutting and sharp foreign bodies should be performed because they can cause severe injuries. The complication rate of sharp foreign bodies may range from <1% to 15%–35% depending on the number and shape of the foreign bodies and the duration of the gastrointestinal contact.^4^ Our patient was 2 years old, which is a high risk age for foreign body ingestion. Foreign body ingestion should be taken into consideration in children within this age group.

While coins are the most common ingested foreign bodies in children in other countries, hooked needles and pins are frequently reported in our country.^5^^,^^6^ Perforation of the gastrointestinal tract and migration of the foreign body to other organs are dangerous complications. Foreign bodies most commonly leading to perforation are fish bones, chicken bones, and needles.^7^ In our case, the foreign body that was ingested and that migrated outside of the lumen was a sewing needle.

The general indications for removing ingested foreign bodies in children are **sharp** foreign bodies in the stomach, disc batteries and toxic FB, foreign bodies in the esophagus, FB longer than 4 cm and wider than 2 cm, FB present for more than 2 to 6 weeks in the stomach and one week in the duodenum.^8^ Sharp objects shall be removed immediately by endoscopy if they are present in the esophagus, stomach or proximal duodenum. If a sharp foreign body has passed beyond duodenum then it may be observed for up to three days. After three days a sharp foreign body shall be removed by endoscopy, laparoscopy or surgery. In the present case, the ingested sewing needle was checked by periodic plain X-ray examinations in an external center; however, the foreign body was not spontaneously expelled for approximately 3 months. This long time period, as well as the fact that the foreign body could be palpated but not observed by rectal examination, suggested that it had migrated outside of the lumen. Then, the foreign body was removed by a mini-laparotomy procedure. In the literature, a limited number of migration cases related to foreign body ingestion in children have been reported.^9^^-^^16^ The ages of these patients, 5 males and 4 females, ranged between 7 months and 16 years. The anatomic areas where migration occurred were liver (n: 3), diaphragm (n: 2), intraabdominal space (n: 2) and deep neck area (n: 2). Abscess developed as a complication in 2 patients who had deep neck migration and in 1 patient who had liver migration.^9^^,^^12^^,^^15^ A variety of foreign bodies were reported (needle/pin, nail, dental braces, marble, pencil, grass blade). Some of these cases were treated by an endoscopic procedure (n:4), while surgical treatment was administered for others (n:5).

## CONCLUSION

Gastrointestinal sharp foreign bodies in children, which has not passed after a few days of observation should be removed or referred to an appropriate center. In cases of foreign body ingestion by children, radiological follow-up may not be sufficient and physical examination of the abdomen may give important information. Foreign bodies that are suggested to have migrated outside of the lumen should be considered for removal by an endoscopic or surgical procedure, without delay, to prevent traumatic complications.

## Author’s contributions:

**MNC, MD, BD, ID, OK:** Substantial contributions to conception and design, or analysis and interpretation of data.

**MNC, MD, BD, ID, OK:** Drafting the manuscript and revising it critically for important intellectual content.

**MNC, OK:** Final approval of the version to be published.
